# Developing a Novel Agrochemical-Based MOF: A Multifunctional Platform with Herbicidal and Antibacterial Activities

**DOI:** 10.1021/acsami.4c17237

**Published:** 2025-01-07

**Authors:** MCarmen Contreras, Pablo Salcedo-Abraira, Andoni Zabala-Lekuona, Antonio Rodríguez-Diéguez, Sara Rojas

**Affiliations:** † Department of Inorganic Chemistry, Faculty of Science, 16741 University of Granada, Av. Fuentenueva s/n, 18071 Granada, Spain; ‡ Department of Applied Chemistry, Faculty of Chemistry, Euskal Herriko Unibertsitatea (UPV/EHU), 20018 Donostia, Spain

**Keywords:** metal−organic frameworks, agriculture, herbicide, antibacterial, multifunction, controlled release

## Abstract

Excessive and uncontrolled application of agrochemicals has resulted in contamination of terrestrial and aquatic environments. In the past decade, metal–organic frameworks (MOFs) have been studied as agrochemical release systems to enhance efficiency while reducing the leaching of toxic molecules to the environment. In this work, we take a further step and use organic agrochemicals as linkers in the preparation of MOFs, which we have called AgroMOFs. Particularly, we have prepared a novel 2D framework, named GR-MOF-20, based on the flexible herbicide glyphosine (H_5_Gly) and the widely used antibacterial and fungicide Cu^2+^. After complete characterization by single-crystal and powder X-ray diffraction and high-performance liquid chromatography (HPLC), a fascinating transformation of the structure to a novel 3D-MOF (GR-MOF-21) after in water is observed, together with a burst degradation (86.0 ± 3.2% after 72 h). Considering the bioactivity of its building blocks (H_5_Gly and Cu^2+^), the combined herbicidal and antibacterial activities of GR-MOF-21 were demonstrated. Aqueous solutions prepared with GR-MOF-20 (450 mg L^–1^) demonstrated a better inhibition effect (21.7 ± 2.9% after 7 days) in the germination of seeds of the invasive weed than the free H_5_Gly (13.3 ± 2.9%). Further, the selectivity of GR-MOF-21 was tested against a nontarget plant (wheat, ). Finally, GR-MOF-21 showed an important antibacterial activity against and , achieving the results obtained with Cu^2+^ solutions. These observations demonstrated that the use of herbicide and antibacterial units in the MOF construction is an efficient approach to controlling bacteria and weed plants without damaging wheat crops.

## Graphical abstract

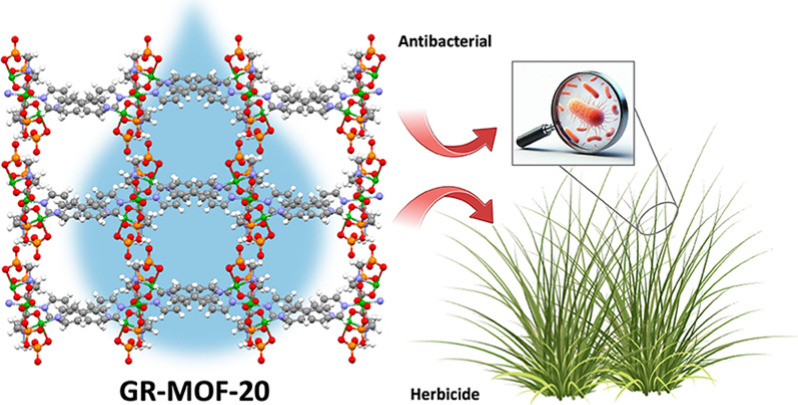

## 1 Introduction

The convergence of population growth and climate change threatens food security on a worldwide scale. Current trends in population growth suggest that global food production is unlikely to satisfy future demand, as predicted. According to the United Nations Fund for Population Activities (UNFPA) report,^1^ the world population is projected to reach 9.7 billion in 2050 and 10.4 billion by 2100. Further, the Intergovernmental Panel on Climate Change indicates that rising temperature, drought, floods, desertification, and extreme weather will severely affect agriculture.^2^ Thus, modern agriculture is confronted with the simultaneous task of feeding an increasing amount of population without decreasing or improving the quality and safety standards. In order to accomplish these significant objectives, the use of agrochemicals (fertilizers, pesticides, plant hormones, etc.) becomes inevitable to ensure quality and high yields. However, only ca. 10% of the agrochemicals applied using traditional methods reach their target,^3,4^ requiring several doses to maintain their desired effect (active concentration), frequently causing the contamination of terrestrial and aquatic environments. As a consequence, a well-established pollution pattern is observed, with a major pollution peak occurring after a few days or weeks following the application of the agrochemicals.^5^ The detection and quantification of agrochemicals in water sources together with their potential environmental health risks have been extensively documented all over the world. According to some reported investigations, among the 13 most frequent pesticides, 10 are herbicides.^6^ For example, the presence of concentrations potentially toxic of glyphosate, one of the most widely used nonselective herbicide, has been reported in the water bodies of Canada (6.079 μg L^–1^),^7^ United States of America (328 μg L^–1^),^8^ Mexico (1.42 μg L^–1^),^9^ Swiss (2.6 μg L^–1^),^10^ and Colombia (from 201 to 2.777 μg L^–1^).^11^ Further, pesticide exposure through water ingestion reduces body immunity, disrupts hormone balance, triggers reproductive issues, poses carcinogenic effects, and reduces intelligence, particularly in children at the body development stage.^12^ Consequently, considering this global environmental problem, there is great interest in the development of more efficient and safe agrochemicals.

A novel trend in agrochemical production is the design of slow agrochemical release systems to enhance efficiency while reducing agrochemicals leaching to the environment.^13^ Controlled agrochemical release systems are more versatile and provide optimum active ingredient (AI) levels throughout the crops’ entire growing cycle, with a wide range of longevities, from 1 month up to 1.5 years.^14^ In recent decades, several smart composites are being designed as carriers of agrochemicals, such as encapsulated agrochemicals in different matrix, i.e., poly­(2-(dimethylamino)­ethylmethylacrylate)-*b*-poly­(ε-caprolactone) nanoparticles as carrier of tebuconazole,^15^ flexible eugenol-loaded polyethylene glycol-based particles,^16^ and sodium diisooctyl succinate sulfonate vesicles as carriers of the insecticide nicotine hydrochloride.^17^ Among all of the proposals, metal–organic frameworks (MOFs) highlight as agrochemical carriers.^18^ MOFs are considered a unique class of porous coordination polymers comprising inorganic nodes (e.g.*,* atoms, clusters, and chains) and organic polydentate linkers (e.g.*,* carboxylates and phosphonates) that assemble into multidimensional porous periodic lattices.^19^ MOFs present several properties that make them exceptional candidates in the preparation of novel formulation in the agroindustry: (i) versatile hybrid compositions, which allow multiple combinations; (ii) a large specific surface area associated with exceptional sorption capacities; (iii) some are based on nontoxic precursors; (iv) some are synthesized at a large scale and are commercially available. The three main strategies proposed for the application of MOFs in the agroindustry are as adsorbents of agrochemicals in water remediation, as sensors in the determination of agrochemicals in water or food, and finally as agrochemical-controlled release systems. In this regard, the use of MOFs in the delivery of agrochemicals has become a hot topic in the past few years (with 88% of reports published in the last 4 years, according to Web of Science, September 2024, “Metal-Organic Frameworks” “agriculture” “controlled release”). In the first example dated from 2015, Anstoetz et al. described the use of an oxalate-phosphate-amine-based MOF as a microbially induced slow-release N and P fertilizer.^20^ Recent examples are the controlled release of the herbicide 2,4-dichloropheoxycetic acid (2,4-D) from UiO-66 and UiO-66-NH_2_ ^21^ or the MOF-on-MOF materials based on UiO-66 and ZIF-8 in the loading of the insecticide dinotefuran and the fungicide tebuconazole.^22^ Considering the compositional versatility of MOFs, we want to take a further step and use organic agrochemicals as linkers in the preparation of MOFs, what we have called AgroMOFs.^23^ AgroMOFs are considered attractive formulations for target multiple release of agrochemicals: (i) a simple one-pot synthesis is needed, avoiding multiple steps to load the active ingredient in the MOF’s porosity; (ii) one formulation is active against a variety of pests which is economically appealing; and (iii) all degradation products are active, reducing the environmental damage. In our first work, Cu^2+^ and the naturally occurring herbicide glufosinate were used as the ligand in the construction of the first AgroMOF (named GR-MOF-7). Glufosinate is a contact herbicide with limited translocation, making it effective primarily on annual weed species with lower activity on larger weeds. Interestingly, other herbicides, such as glyphosine, are active against a wide variety of weeds, inhibiting fibber production. Thus, in this work, we selected for the first time in the construction of an AgroMOF, the flexible herbicide glyphosine (H_5_Gly), which has four potential metal-binding sites (two phosphonate, one amine, and one carboxylic groups), which will facilitate the MOF formation. H_5_Gly is a common herbicide which causes chlorosis of emerging leaves, and it is normally used in cereals (maize, wheat) or sugar cane crops which represent the world’s most produced crops.^24,25^ It should be noted that H_5_Gly has never been loaded in a carrier for its application as an agrochemical release system. Thus, here we report the synthesis and characterization of a novel 2D-MOF (named GR-MOF-20) based on the herbicide H_5_Gly and the widely used bactericidal and fungicidal agent copper and a novel 3D-MOF (named GR-MOF-21) resulting after a phase transformation of the GR-MOF-20 compound. After complete characterization and scale up of the GR-MOF-20 synthesis, the multiple bactericide and herbicide effect was tested and compared with its precursors [Cu­(NO_3_)_2_·3H_2_O, free H_5_Gly, etc.]. This work highlights the importance of the materials’ conceptual selection, focusing on a meticulous choice of ligands (agrochemicals) and cations (bioactive) in the preparation of multifunctional agrochemicals.

## 2 Methodology

### 2.1 Synthesis of GR-MOF-20

26.3 mg (0.1 mmol) amount of H_5_Gly and 48.3 mg (0.2 mmol) of Cu­(NO_3_)_2_·3H_2_O were dissolved in 2 mL of distilled water. In a separate vial, 31.2 mg (0.2 mmol) of 4,4′-bipyridine (4,4′-Bipy) were dissolved in 2 mL of ethanol. Then, H_5_Gly and Cu^2+^ solution was mixed over the 4,4′-Bipy solution. The final solution in a closed vial was introduced in an oven at 95 °C for 24 h. After the reaction time, suitable blue crystals for single-crystal X-ray diffraction were obtained and filtered off under vacuum using a filter plate.

Different techniques were used to validate the synthesis of the new compound. In this regard, Fourier transform infrared (FTIR) spectrum confirms the presence of coordinated glyphosine and 4,4′-Bipy as the characteristic bands at 1727 and 1590 cm^–1^ that correspond to ν­(C=O) and ν­(C=N) from the free linkers are completely shifted to 1627 and 1612 cm^–1^, respectively (Supporting Information-SI, Figure S1). The chemical composition of GR-MOF-20 was confirmed by elemental analysis (N, C, and H) and inductively coupled plasma (ICP, P and Cu), attributing the remaining atoms to O. Theo. (%): N (6.78), C (27.14), H (4.55), P (9.99), and Cu (15.38). Exp. (%): N (7.14), C (28.05), H (5.68), P (9.81), and Cu (15.71).

The molecular formula of the synthesized compound is [Cu_3_(H_2_Gly)_2_(4,4′-Bipy)_2_]·12H_2_O or [Cu_3_(NP_2_C_4_O_8_H_8_)_2_(C_10_H_8_N_2_)_2_]·12H_2_O.

### 2.2 Scaled-Up Synthesis of GR-MOF-20

The synthesis was scaled up by 20. Hence, 526.2 mg (2 mmol) of H_5_Gly and 966.5 mg (4 mmol) of Cu­(NO_3_)_2_·3H_2_O were dissolved in 40 mL of distilled water. In a separate vial, 624.8 mg (4 mmol) of 4,4′-Bipy were dissolved in 40 mL of ethanol. Then, H_5_Gly and Cu^2+^ solution was mixed over the other one in a round-bottom flask glass. The final solution was refluxed at 95 °C with stirring for 24 h. The powder obtained was filtered under vacuum. 976 mg of compound was obtained (yield = 78.76% calculated based on H_5_Gly).

### 2.3 Synthesis of GR-MOF-21

When crystals of GR-MOF-20 coordination polymer are immersed in tap water, the formation of a new 3D-MOF, GR-MOF-21, is observed after 15 min and completed after 5 h (vide infra). The synthesis was controlled by a solution of 50 mg (0.04 mmol) of GR-MOF-20 with 2 mL of calcium acetate (Ca­(CH_3_COO)_2_) solution (0.05 M). After 24 h, the phase transformation was confirmed by powder X-ray diffraction (PXRD), and the GR-MOF-21 material was obtained (yield = 63.22%, calculated based on GR-MOF-20 based on 4,4′-Bipy).

The molecular formula of the synthesized compound is [Cu_5_(Gly)_2_(4,4′-Bipy)_4_]·*n*H_2_O (*n* = 18–20) or [Cu_5_(NP_2_C_4_O_8_H_6_)_2_(C_10_H_8_N_2_)_4_]·*n*H_2_O (*n* = 18–20).

### 2.4 Stability Studies

The chemical stability of GR-MOF-20 was determined in tap water at room temperature by measuring the release of 4,4′-Bipy and H_5_Gly by high-performance liquid chromatography (HPLC) and the release of P by inductively coupled plasma mass spectrometry (ICP-MS), respectively. The structural stability was also determined by measuring the PXRD patterns at different times. 20 mg of GR-MOF-20 was suspended in 20 mL of tap water under stirring for 7 days at room temperature. At different incubation times (0.25, 1, 2, 5, 7, 24 h, and 2, 3, 4, and 7 days), the suspensions were centrifuged (4000 rpm, 20 min); an aliquot of 10 mL was extracted, and the same volume of water was added in a way to keep sink conditions, while the solids were filtered and analyzed by PXRD. All kinetic studies were carried out in triplicate (*n* = 3).

### 2.5 Herbicidal Activity

The herbicidal activity was studied in terms of seed germination. Seeds of (*L. multiflorum*, Italian ray-grass), selected in this study as a model invasive weed, were purchased from an authorized dealer with certification, Batlle. The average germination rates of the plant seeds were greater than 93.8%. Seeds were kept in a dry place in the dark at room temperature before use.

First, the minimum active concentration of H_5_Gly against was determined. Aqueous solutions (20 mL) of H_5_Gly with different concentrations (100, 200, 250, 300, and 350 mg L^–1^) were tested. In parallel, a negative control with only water was performed. 50 mL beakers (12.56 cm^2^ surface area) were used for each different concentration, with a total of 20 seeds per recipient. germination was studied for 7 days. All experiments were performed with an average day/night photoperiod of 15–9 h and a temperature of 25–15 °C. The number of germinated seeds was counted to obtain the germination rate.

Once the active concentration of H_5_Gly (200 mg L^–1^) was determined, the activity of GR-MOF-21 was studied against ray-grass. One has to consider the results obtained in the aqueous stability of GR-MOF-20. Here, although we are preparing aqueous solutions with GR-MOF-20, the activity is related to GR-MOF-21 (formed only after 15 min of suspension). 20 mL of an aqueous solution of GR-MOF-20 (450 mg L^–1^) was added to a beaker containing 20 seeds following the same procedure as previously described. In parallel, a negative control with water was performed. All tests were carried out in triplicate (*n* = 3).

#### 2.5.1 Selectivity

The selectivity and safety of GR-MOF-20 were tested against the nontarget plant wheat () supplied by Agrointec (Almería, Spain). Before use, the seeds were stored in a dry dark place at room temperature. Initially, wheat seeds were sterilized by washing for 30 s with ethanol, rinsed with distilled water, washed for another 30 s with 0.1 M HCl, and rinsed again with distilled water. Then, the seeds were germinated in a wet paper under dark conditions at 22 °C, obtaining a high rate of germination (*ca.* 90%) after 3 days of incubation. Wheat seedlings were treated with GR-MOF-20 suspensions of different concentrations (0, 440, 560, 670, and 780 mg L^–1^). It is important to highlight that during the selectivity tests, we go a further step, and the almost doubled (1.75-fold) concentration of GR-MOF-20 was also tested. Plants were kept under ambient conditions of humidity under dark at 22 °C. After 5 days, dried plant weight (100 °C for 24 h) was measured to evaluate the potentially toxic effect. The results of the different assays are represented as the mean ± standard deviation (SD). Each experiment was performed at least three times (*n* ≥ 3). Measurements were analyzed statistically using a one-factor ANOVA test to determine the significance between the average of the different concentrations.

### 2.6 Antibacterial Studies

The Gram-negative () (CECT 101) and () (CECT 126) bacteria were used as reference strains for the antibacterial activity tests. Bacteria were grown, and antibacterial tests were performed according to the supplier recommendation. The microorganisms were preserved at −20 °C in glycerol (25% v/v) until their use. Reactivation was performed in tryptic soy broth (TSB; composed by 17 g L^–1^ casein peptone, 3 g L^–1^ soy peptone, 5 g L^–1^ NaCl, 2.5 g L^–1^ dipotassium phosphate, 2.5 g L^–1^ D­(+)-glucose (dextrose) monohydrate, pH = 7.3 ± 0.2) at 37 and 25 °C for and , respectively. Innocuous of approximately 10^8^ cells·mL^–1^ bacteria were prepared in 1/500 TSB.

For the antibacterial experiments, the previously set active concentration of GR-MOF-20 (450 mg L^–1^) was used and the corresponding concentrations of 120, 200, and 275 mg L^–1^ for the different precursors separately (4,4′-Bipy, H_5_Gly, Cu­(NO_3_)_2_·3H_2_O, respectively). Each compound was dissolved in 2 mL of inoculum in 24-well plates and carried out in triplicate (*n* = 3). After 24 h of incubation under stirring, the bacterial viability was evaluated under suspension [by colony-forming units (CFUs) and fluorescein diacetate staining (FDA)] methods.

#### 2.6.1 CFUs

Bacteria aliquots were placed in sterile 96-well plates in 10-fold serial dilutions in phosphate-buffered solution. Three 20 μL drops of each dilution were placed in Petri dishes containing TSB agar medium and, after 24 and 48 h incubation at 37 and 25 °C for and , respectively, CFUs were counted.

#### 2.6.2 FDA

A nonfluorescent compound hydrolyzed by esterases in fully functional cells to a green fluorescent compound (fluorescein) was here used as the indicator for bacteria enzymatic activity determination. Thus, a liquid fraction was analyzed in 96-well microplates by mixing 5 μL of FDA (2 mg mL^–1^) in dimethyl sulfoxide, with 195 μL of bacterial suspension in each well. The plate was incubated for 45 min with continuous readings every 15 min (λ_ex_ = 485 nm; λ_em_ = 538 nm) using a plate reader NanoQuant (Tecan Trading AG, Switzerland). The possible fluorescence interference of the culture medium was also checked. Each sample was measured nine times, disclosing the outcomes as an inhibition percentage.

## 3 Results and Discussion

### 3.1 Synthesis and Structural Description of GR-MOF-20

A novel 2D AgroMOF based on copper was prepared by heating a mixture of H_5_Gly, 4,4′-Bipy, and copper nitrate in a mixture of water and ethanol at 95 °C for 24 h. In all cases, big rectangular blue prism crystals suitable for single-crystal X-ray diffraction (SCXRD) were isolated (1.48 ± 0.77 × 0.82 ± 0.35 μm^2^, *n* = 56; SI, Figure S2). The structure of GR-MOF-20 consists of two crystallographically independent copper metal centers. Cu1, with a coordination number of five, has a square-based pyramidal geometry (Figure 1a). In the equatorial plane, Cu1 is coordinated to two O atoms, one from a phosphonate and one from a carboxylate, to a ternary amino group of the same H_2_Gly ligand, and to one N from a 4,4′-Bipy linker. On the axial axis, Cu1 coordinates with one phosphonate belonging to H_2_Gly. Cu2 has a coordination number of six with octahedral geometry (Figure 1b). The equatorial plane is formed by the coordination of two phosphonates from two different H_2_Gly groups and two 4,4′-Bipy linkers. On the axial axis, Cu2 atoms are coordinated to two carboxylates from two H_2_Gly ligands. The Cu–O bond distances of the coordinated phosphonate groups in the equatorial plane range from 1.93761(4) to 1.98373(5) Å. The Cu–N bond distances in the equatorial plane range from 1.99161(4) to 2.06081(4) Å. On the axial axis, the Cu–O bond distances range from 2.34317(5) to 2.42904(5) Å due to the well-stablished Jahn–Teller effect in divalent copper metal atoms.^26^ The copper metal centers expand to form 1D chains linked by H_2_Gly ligands, which are further expanded to form a 2D framework by 4,4′-Bipy. The 2D layers interact via hydrogen bonding between the protonated phosphonate groups, which results in 1D pores of size 9.77 × 8.27 Å^2^ in the [0,0,1] direction (Figure 1c). CCDC reference number for the structure is 2384611.

**Figure 1:**
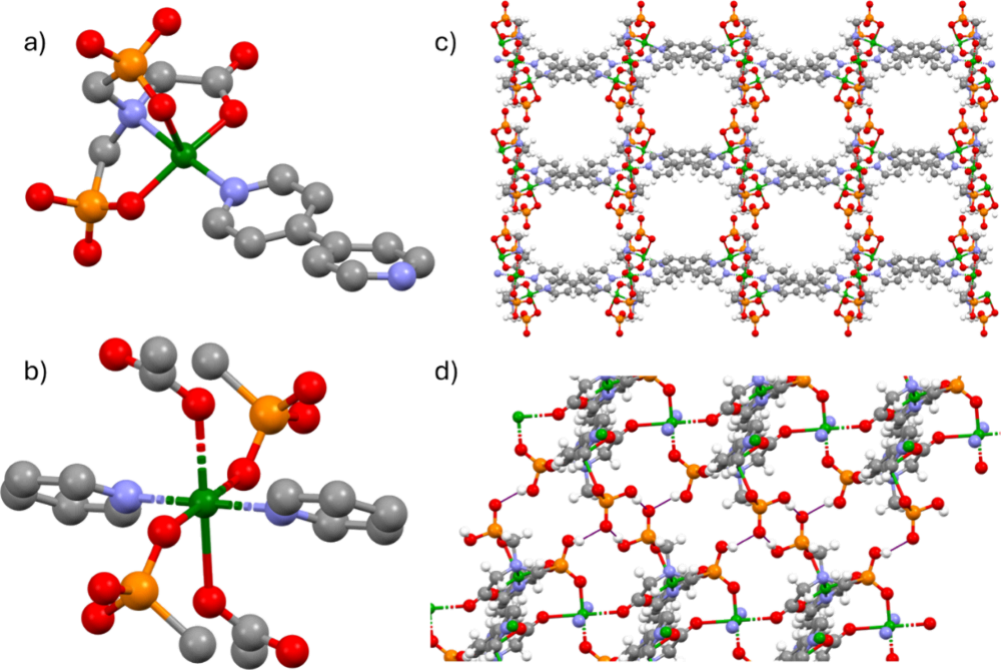
Crystal structure of GR-MOF-20 showing (a) Cu1 and (b) Cu2 coordination geometries. (c) Channels along the [0,0,1] direction. (d) Hydrogen bonding network between layers (in purple dash lines). H_2_O molecules and hydrogens in sections a and b have been omitted for clarity. Color code: O atoms are in red, C in gray, N in blue, P in orange, Cu in green, and H in white.

Notably, the synthesis of GR-MOF-20 was successfully scaled up by 20, obtaining *ca.* 1 g (78.8% yield) of solid in a single reaction batch avoiding pressure by using reflux conditions (see Section 2.2). The characteristic crystalline phase of GR-MOF-20 was identified by PXRD in the scaled-up bulk sample by comparing both the location and intensity of the main Bragg reflections with those of the crystalline structure resolved by SCXRD (SI, Figure S3). To check the phase purity, Le Bail fitting was carried out using the unit cell parameters of the GR-MOF-20 (SI, Figure S4).

### 3.2 Stability Studies

Previous to the use of any agrochemical, it is imperative to fully characterize it, including the evaluation of their thermal and chemical stabilities in the working media. Thermogravimetric analysis (TGA) shows an initial weight loss (11.84%) from room temperature to 130 °C associated with the departure of eight water molecules (SI, Figure S5). Then, at around 200 °C, the material starts to degrade with the decomposition of the ligands. The final residue was identified as a mixture of Cu_2_O_7_P_2_, CuO_6_P_2_, and Cu_3_O_8_P_2_ (DIFFRAC.EVA, Bruker, SI, Figure S6). Further, considering that agrochemicals are normally sprayed as an aqueous solution or suspension in the fields and/or the release can be driven by humidity or rain, the stability of GR-MOF-20 was checked in tap water. Particularly, glyphosine is normally sprayed as a 0.1 M aqueous solution.^25^ The aqueous chemical stability of GR-MOF-20 was investigated in terms of linkers leaching, 4,4′-Bipy was measured by HPLC (Figure 2a, SI Section S3), and H_5_Gly was determined measuring the concentration of P by ICP-MS. It is worth noting that the GR-MOF-20 degradation exhibited two steps. Initially, a burst release is observed with 63.0 ± 2.3% and 82.2 ± 3.6% of 4,4′-Bipy and H_5_Gly after 7 h, respectively, with a subsequent slower release, achieving the plateau after 72 h with 86.9 ± 3.2% and 93.9 ± 2.8% of released 4,4′-Bipy and H_5_Gly, respectively.

**Figure 2:**
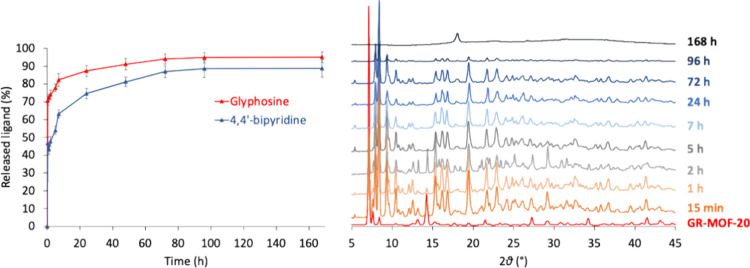
GR-MOF-20 stability in aqueous media monitored by (a) HPLC and (b) PXRD.

Regarding the structural stability, a transformation was observed after 15 min when crystals of GR-MOF-20 were immersed in tap water_,_ resulting in a full formation of a new 3D-MOF GR-MOF-21 after 7 h. Crystal structure of the new MOF comprises square pyramidal Cu­(Gly) units, described for GR-MOF-20 in Figure 1a, interlinked by a 4,4′-Bipy molecule forming a Cu_2_(Gly)_2_(4,4′-Bipy) dimer (Figure 3a). Note here that the agrochemical in GR-MOF-21 is fully deprotonated, while in GR-MOF-20, glyphosine appears as triply deprotonated. The Cu­(Gly) units are further linked through two oxygens of a phosphonate group to two different Cu (Cu3 and Cu4) centers and through one oxygen of the second phosphonate to a Cu2 center (Figure 3b). Cu3 and Cu4 present a distorted octahedral coordination geometry with an equatorial plane formed by two oxygens of two adjacent phosphonates and two nitrogens of different 4,4′-Bipy ligands. Two water molecules are also coordinated axially, with longer Cu–O bonds [distance range 2.421(6)-2.548(5)] as a consequence of the well-known Jahn–Teller effect.^26^ Cu2 metals are found disordered over two positions and present a distorted square pyramidal coordination geometry with two oxygens from two phosphonates and two nitrogens from two 4,4′-Bipy forming the square base and a single water molecule coordinated axially. The overall 3D structure is built by 2D layers formed by the Cu_2_(Gly)_2_(4,4′-Bipy) dimers interlinked by the two octahedrally coordinated coppers (Cu3 and Cu4), which are further expanded into a 3D framework by the square pyramidal copper centers (Cu2), forming 1D channels alongside the [0,1,0] direction (Figure 3c). CCDC reference number for the structure is 2384610.

**Figure 3:**
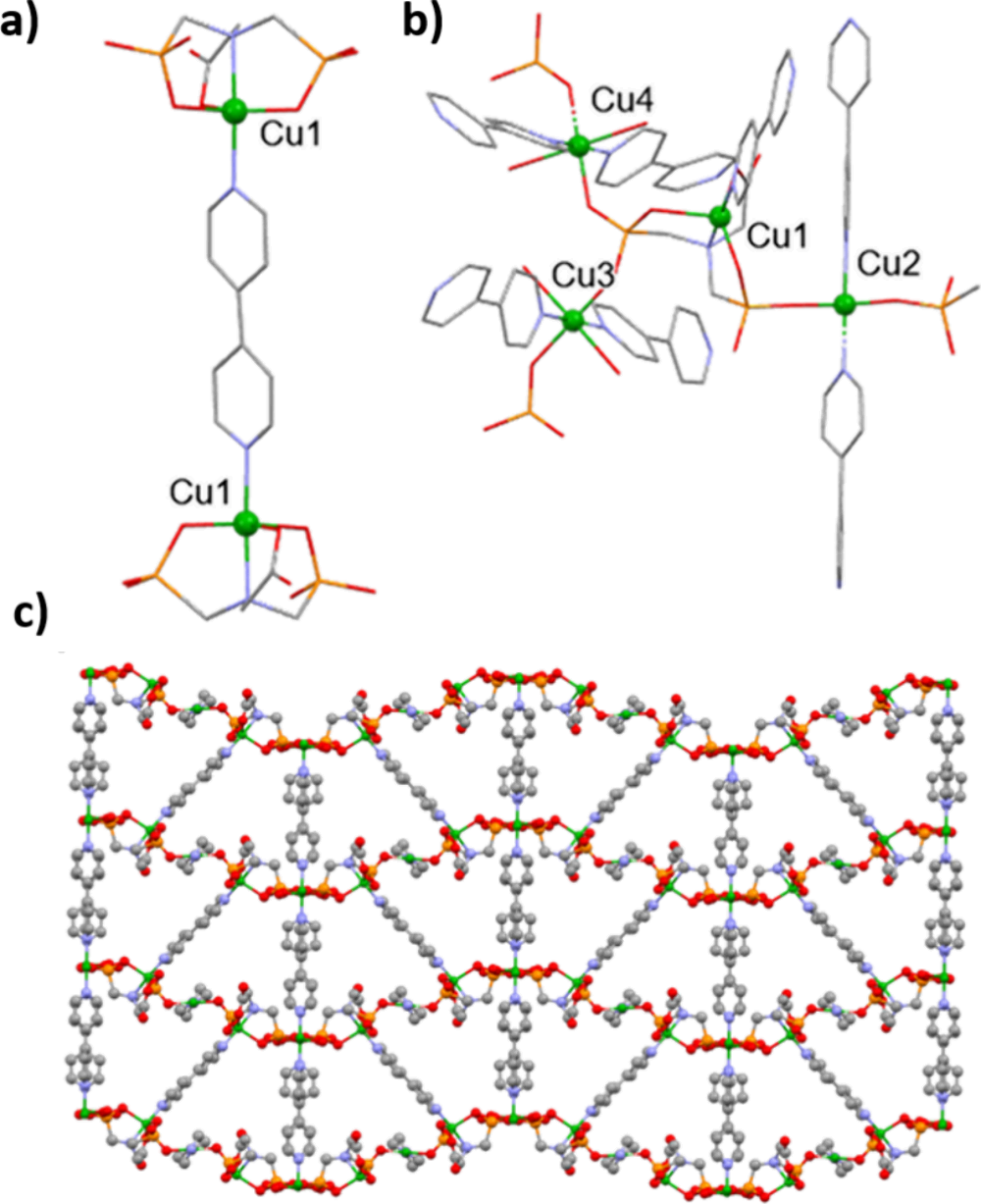
Crystal structure of GR-MOF-21. (a) Coordination of Cu1 and the formation of the dimeric unit; (b) coordination geometry of Cu1, Cu2 Cu3, and Cu4; and (c) packing the [0,1,0] axis showing the triangular pores. Solvated water molecules are omitted for clarity. Color code: O atoms are in red, C in gray, N in blue, P in orange, Cu in green, and H in white.

The large GR-MOF-20 blue needle crystals morphologically changed into slightly smaller blue block crystals during the GR-MOF-20 → GR-MOF-21 transformation. Moreover, the difference between the formula stoichiometries of GR-MOF-20 and GR-MOF-21 suggests that the GR-MOF-20 → GR-MOF-21 transformation occurs through a recrystallization mechanism, requiring the presence of alkaline earth cations in solution. This transformation was also monitored through PXRD using tap water and calcium acetate solution. The PXRD results shown in Figure 2b indicate that most of the transformation occurs within 15 min under tap water, while in the presence of calcium acetate (Figure 4), the transformation develops gradually over a period of 24 h. Note that tap water was used as agrochemicals are normally sprayed using this type of water (not distilled or Milli-Q). Finally, considering the fast transformation to GR-MOF-21 and the initially fast release of the active components, it is not possible to store this material as an aqueous solution; thus, it is preferred to suspend the material in water just before its application in the field as an aqueous suspension.

**Figure 4:**
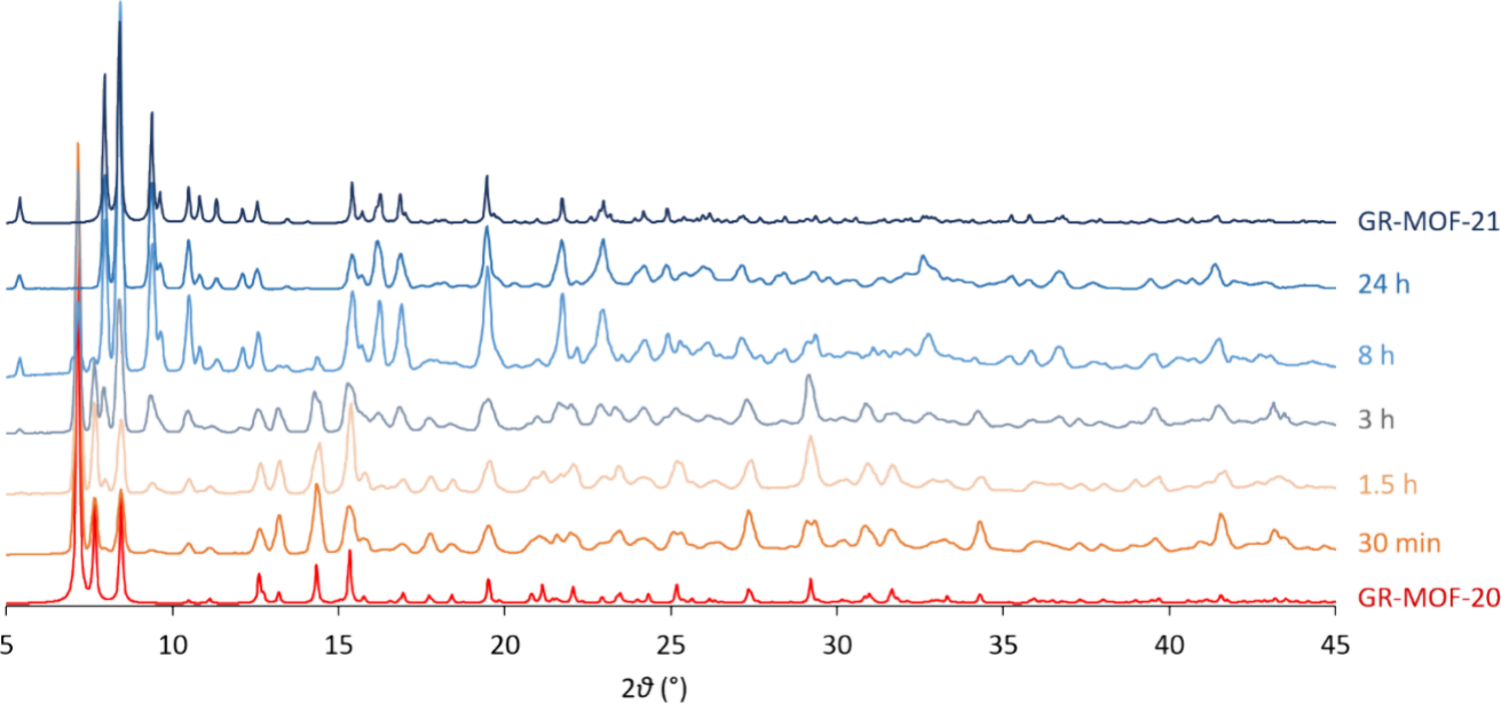
GR-MOF-20 transformation into GR-MOF-21 in Ca­(O_2_CCH_3_)_2_ solution monitored by PXRD.

### 3.3 Herbicidal Activity

In our aim to demonstrate the multifunctional character of GR-MOF-20, the effectiveness of GR-MOF-20 as a herbicide was tested against seeds of an herbaceous invasive grass ( or Italian ray-grass) which is normally found in important crops (i.e., corn).^27,28^ First, the minimum active H_5_Gly concentration was determined by submerging ray-grass seeds in aqueous solutions with different H_5_Gly concentrations (see Section 2.4 for further details). After 7 days, H_5_Gly (200 mg L^–1^) was able to inhibit 13.3 ± 2.9% of seed germination (SI, Figure S8), which was considered the minimum active concentration in this study. When using this concentration, based on the amount of H_2_Gly in GR-MOF-20, the MOF (450 mg L^–1^) was able to inhibit 21.7 ± 2.9% of seed germination after 7 days, showing a significantly (*p* < 0.05) greater herbicide effect than the free H_5_Gly (13.3 ± 2.9%, Figure 5). Note here that although aqueous solutions of GR-MOF-20 are prepared and in agreement with the stability tests conclusion, the activity under suspensions is driven by compound GR-MOF-21 (formed only after 15 min).

**Figure 5:**
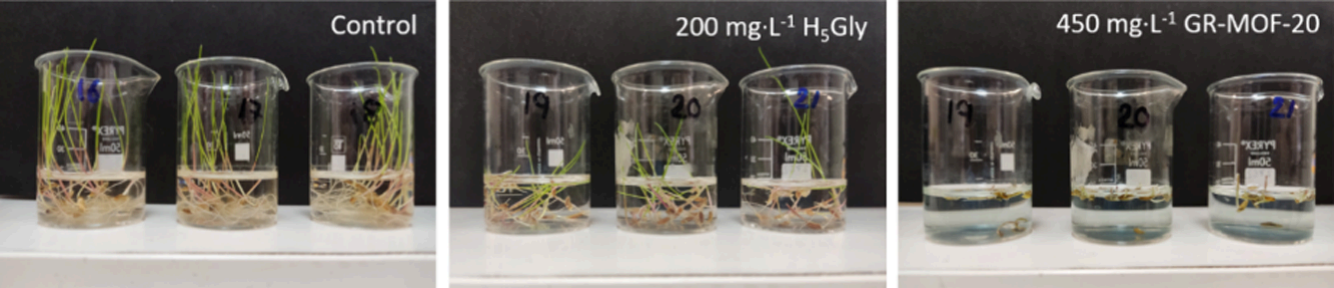
Germinated seeds after being treated with aqueous solutions of H_5_Gly, GR-MOF-20, and water (control) for 7 days.

In an attempt to assess the selectivity of this novel material in crops, GR-MOF-21 was tested against a nontargeted plant (, wheat). No signs of toxicity, evaluated as the seedling dried weight, were observed when treated with 0, 440, 560, 670, and 780 mg L^–1^ of GR-MOF-20 (SI, Figure S9). These observations demonstrated the safety and selectivity of GR-MOF-21, which can be used to control weed plants without damaging wheat crops.

### 3.4 Antibacterial Effect

Since the initial use of Bourdeaux mixture in 1885 for disease control,^29^ a large number of Cu-based antimicrobial compounds have been developed and applied for crop protection. In this work, the combined herbicidal and bactericidal effect of GR-MOF-21 was investigated, evaluating its antibacterial activity against the common foodborne bacteria and the plant pathogen .^30^

The antibacterial activity of GR-MOF-21 was determined by the bacteria viability, following both the CFUs and the microbial enzymatic activity using the FDA hydrolysis assay. In these tests, the same concentration of GR-MOF-20 (450 mg L^–1^) previously found to be active as an herbicidal agent was used. Note here that this concentration of GR-MOF-20 corresponds to 73 mg L^–1^ of Cu^2+^, which is within the range (1–200 mg L^–1^) found in normal soils.^31^ Additionally, the activity of negative (water) and positive controls of the different precursors separately (4,4′-Bipy, H_5_Gly, Cu­(NO_3_)_2_·3H_2_O) at the same concentration at which they are present in the MOF (120, 200, and 275 mg L^–1^, respectively) was performed. GR-MOF-21 showed a high antibacterial effect against both bacteria, with 99.9 ± 1.5% and 97.9 ± 0.8% of enzymatic inhibition after 24 h for and , respectively (Figure 6a,b). In particular, against , 4,4′-Bipy did not let a significant antimicrobial performance (15.1 ± 6.8%), whereas both Cu­(NO_3_)_2_·3H_2_O and H_5_Gly also showed high bacterial inhibition (91.4 ± 2.1% and 86.4 ± 3.2%, respectively), while in the case of , 4,4′-Bipy showed a greater effect on enzymatic inhibition than H_5_Gly (79.8 ± 0.8% and 0.1 ± 16.2%, respectively). As with the other bacteria, Cu­(NO_3_)_2_·3H_2_O also showed a high inhibition (97.0 ± 0.5%) against .

**Figure 6:**
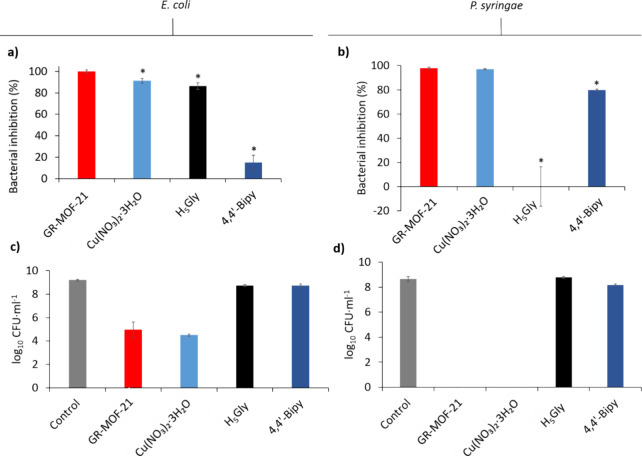
Bacteria viability after 24 h of contact with GR-MOF-21, Cu­(NO_3_)_2_·3H_2_O, H_5_Gly, and 4,4′-Bipy. (a,b) Viability determined by FDA hydrolysis assay. In all cases, values were normalized using the media signal. (c,d) Viability determined by CFUs. The statistical significance with respect to GR-MOF-21 was disclosed as *p < 0.001.

The bactericidal activity of GR-MOF-21 against was confirmed after 24 h of exposure, and the CFU count showed a clear decrease in the number of bacteria compared to the control (Figure 6c), achieving 99.98% bacterial inhibition (Table S2) (4.97 ± 0.67 and 9.20 ± 0.07 log_10_ CFU·mL^–1^ for GR-MOF-21 and water, respectively). Cu­(NO_3_)_2_·3H_2_O had a similar effect than GR-MOF-21 resulting in 99.99% inhibition (4.51 ± 0.09 log_10_ CFU·mL^–1^). This antibacterial activity can be compared with other proposed MOF systems in bactericidal activity (SI, Table S4). On the other hand, H_5_Gly and 4,4′-Bipy did not show as much bactericidal efficacy, resulting in 64.37% inhibition (8.75 ± 0.08 and 8.75 ± 0.12 log_10_ CFU·mL^–1^, respectively) in both cases. When comparing between bacterial strains, the results were even better against (Figure 6d), and GR-MOF-21 and Cu­(NO_3_)_2_·3H_2_O reached 100% inhibition (Table S3) under the parameters studied. 4,4′-Bipy, as in the previous case, showed a lower efficacy (66.67%), and H_5_Gly had no effect on the bacteria. From these results, we can state that the antibacterial effect is mainly due to the presence of Cu^2+^ in the structure, as expected, which presented a high percentage of inhibition (99.99 and 100%) when it was applied freely against both bacteria. The small difference in inhibition between Cu^2+^ and GR-MOF-21 could be due to the difference in the solubility of the two compounds and the slow release of Cu^2+^ from the MOF that shows a degradation of 74.57% at 24 h.

## 4 Conclusions

In this work, following a simple method, a new AgroMOF was synthesized and characterized. GR-MOF-20 was prepared by selecting an active ingredient as the organic ligand with different functional groups capable of interacting with Cu^2+^. Low-polluting solvents were used for the synthesis, which obtained a great yield. XRD, FTIR, SEM, and TGA were used to demonstrate its properties, and its stability was tested in tap water showing a transformation to a novel 3D-MOF (GR-MOF-21) after 15 min and 74.57% of precursor release in the first 24 h. GR-MOF-21 demonstrated herbicidal capacity and showed better results in the percentage of inhibition in germination compared to the free agrochemical. Furthermore, its effect on a nontarget crop was verified by testing different concentrations that did not produce obvious signs of toxicity. On the other hand, when its minimum effective concentration for use as an herbicide was established, the effect of this concentration on the and bacteria was tested. With the results obtained, we can conclude that the compound has bactericidal activity against both bacteria under the conditions tested since the population of the microorganisms was reduced by 99.98 and 100% in 24 h, equivalent to a decrease in CFU of ca. 5 log_10_.^32^

## Supplementary Material



## Notes

Materials and methods, physicochemical characterization, and herbicidal and antibacterial results of GR-MOF-20 (PDF)
