# Supplementary material for: Developing a Novel Agrochemical-Based MOF: A Multifunctional Platform with Herbicidal and Antibacterial Activities
Source: ACS Appl Mater Interfaces. 2025 Jan 7;17(2):4147–54. doi: 10.1021/acsami.4c17237 (PMC12305483; doi:10.1021/acsami.4c17237)
Supplement: Supplementary file 1 [file am4c17237_si_001.pdf]

Supporting Information

Developing a novel agrochemical-based MOF: a multifunctional platform with herbicidal and antibacterial activity

MCarmen Contreras,[a] Pablo Salcedo-Abraira,[a] Andoni Zabala-Lekuona,[b] Antonio Rodríguez-Diéguez,[a,*] Sara Rojas[a,*]

[a] Department of Inorganic Chemistry, Faculty of Science, University of Granada. Av. Fuentenueva s/n, 18071 Granada, Spain.
[b] Department of Applied Chemistry, Faculty of Chemistry, Euskal Herriko Unibertsitatea (UPV/EHU), 20018, Donostia, Spain.

*srojas@ugr.es, *antonio5@ugr.es

Table of content

S1. Materials and Methods 2
S2. GR-MOF-20 characterization 4
S3. Stability studies 7
S4. Herbicide activity 8
S5. Evaluation of the antibacterial activity 9
S6. References 11

S1. Materials and methods

All reactants were commercially obtained and used without further modification. Glyphosine (H_5Gly, ≥98%) was obtained from Sigma-Aldrich. 4,4'-Bipyridine (4,4'-Bipy, >98%) was obtained from TCI. Copper nitrate trihydrate ($Cu(NO_3)_2\cdot 3H_2O$) (>99.5%) and calcium acetate monohydrate ($Ca(CH_3COO)_2\cdot H_2O$) (99.0%) were obtained from Sigma-Aldrich. Ethanol (EtOH, 96%) and acetonitrile (ACN, HPLC grade) were obtained from VWR. Phosphoric acid (H_3PO_4, 85%) was obtained from Merck. Hydrochloric acid (HCl, 36.5-38%) was obtained from Scharlab. The antibacterial activity was evaluated in *Escherichia coli* (*E. coli*, CECT 101) and *Pseudomonas syringae* (*P. syringae*, CECT 126) strains, purchased at Colección Española de Cultivos Tipo (CECT). For the microbiology tests, fluorescein diacetate (FDA, Sigma-Aldrich), tryptic soy broth (TSB, Scharlab), agar agar BAC (labkem) and phosphate-buffered solution (PBS, Millipore) were used.

Fourier transformed infrared (FTIR) spectra were measured in solid state on a Bruker Tensor 27 FT-IR in the range of 4000 to 400 cm^{-1}, and Opus software was used as data collection program. Routine X-ray powder diffraction (XRPD) patterns were collected on a BRUKER D8 DISCOVER diffractometer equipped with a PILATUS3R 100K-A detector and using Cu Kα radiation (λ = 1.5406 Å). The XRPD patterns were registered with a 2θ range from 5 to 45° with a step size of 0.02° and scan rate of 30 s *per* step. Thermogravimetric analyses (TGA) were carried out in a thermogravimetric analyzer mod. TGA/DSC1 METTLER-TOLEDO (Columbus, OH, USA) with a general heating profile from 25 to 950 °C with a heating rate of 5 °C·min^{-1} under air using a flux of 100 mL·min^{-1}. Elemental analyses (EA) were carried out on a Thermo Scientific analyzer model Flash 2000. Scanning electron microscopy (SEM) was carried out using a Hitachi S510 microscopy at 25 kV coupled with a SE detector of 7 nm at 25 kV at Centro de Instrumentación Científica (CIC), University of Granada. Inductively coupled plasma mass spectrometry (ICP-MS) was performed in a spectrometer Nexion 300D (Perkin Elmer, Waltham, MA, USA) at Servicios Centrales de Apoyo a la Investigación (SCAI), University of Málaga.

S1.1 Single crystal structure determination

For GR-MOF-20 and GR-MOF-21, single crystal X-ray diffraction data were collected at 296 K on a Bruker D8 Venture Mo Kα (λ = 0.71073 Å) equipped with a PHOTON 3 detector and an Oxford cryosystem. Data were collected and processed using APEX III and V software. Adsorption correction was applied using SADABS software by empirical methods measuring symmetry equivalent reflections at different azimuthal angles. All structures were solved using the SHELXT program and refined using least squares refinement methods on all F^2 values as implemented within SHELXL.[1,2] Both SHELXT and SHELXL were operated through the Olex2 (v1.5) interface.[3] All atoms were refined anisotropic and atomic displacement parameters were refined with suitable restraints or constraints applied to keep them physically reasonable. Hydrogen atoms were placed in calculated positions and refined with idealised geometries and assigned fixed occupancies and isotropic displacement parameters.

Table S1. Crystallographic data parameters

Identification code	GR-MOF-20	GR-MOF-21
Empirical formula	$C_{28}H_{56}Cu_3N_6O_{28}P_4$	$C_{48}H_{84}Cu_5N_{10}O_{37}P_4$
Formula weight	1239.28	1834.83
Temperature/K	296.15	296.15
Crystal system	monoclinic	orthorhombic
Space group	$P2_1/c$	*Pcca*
a/Å	12.3169(3)	32.8297(11)
b/Å	24.7018(6)	11.0205(3)
c/Å	9.0995(2)	22.2400(8)
α/°	90	90
β/°	110.4180(10)	90
γ/°	90	90
Volume/Å^3	2594.58(11)	8046.4(5)
Z	2	4
ρ_{calc}g/cm^3	1.586	1.515
μ/mm^{-1}	1.431	1.469
F(000)	1274.0	3772.0
Crystal size/mm^3	0.08 × 0.03 × 0.024	0.15 × 0.12 × 0.11
Radiation	MoKα (λ = 0.71073)	MoKα (λ = 0.71073)
2Θ range for data collection/°	5.054 to 52.746	4.452 to 52.74
Index ranges	-14 ≤ h ≤ 15, -28 ≤ k ≤ 30, -11 ≤ l ≤ 11	-40 ≤ h ≤ 41, -13 ≤ k ≤ 13, -26 ≤ l ≤ 27
Reflections collected	14349	53926
Independent reflections	4960 [R_{int} = 0.0206, R_{sigma} = 0.0231]	8149 [R_{int} = 0.0642, R_{sigma} = 0.0289]
Data/restraints/parameters	4960/0/314	8149/980/683
Goodness-of-fit on F^2	1.050	1.104
Final R indexes [I>=2σ (I)]	R_1 = 0.0329, wR_2 = 0.0836	R_1 = 0.0754, wR_2 = 0.2042
Final R indexes [all data]	R_1 = 0.0359, wR_2 = 0.0851	R_1 = 0.0908, wR_2 = 0.2151
Largest diff. peak/hole / e Å^{-3}	0.40/-0.35	1.06/-0.48

S2. GR-MOF-20 characterization

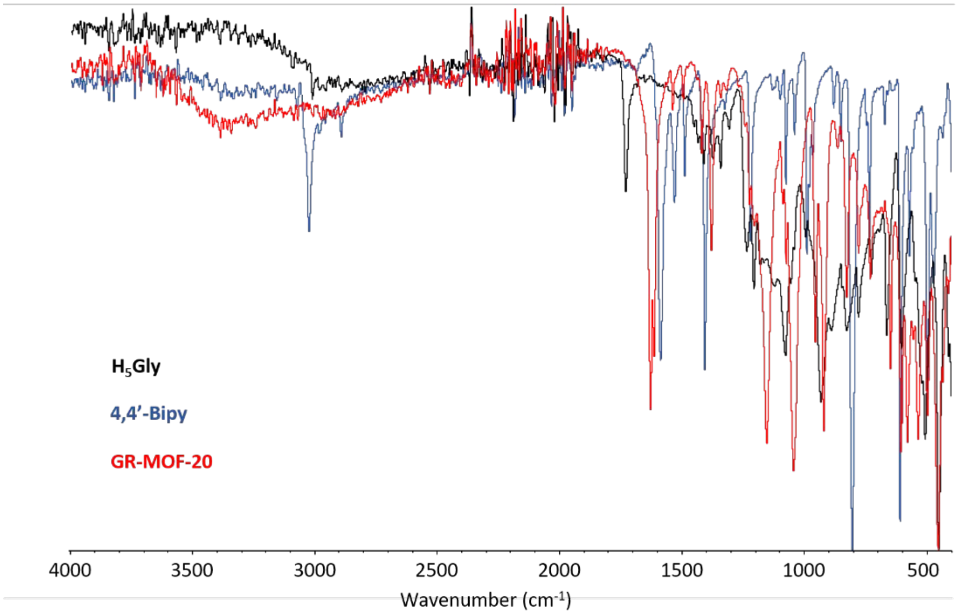

Figure S1. FTIR spectra of GR-MOF-20 (red) compared with free H_5Gly (black) and 4,4’-Bipy (blue) linkers.

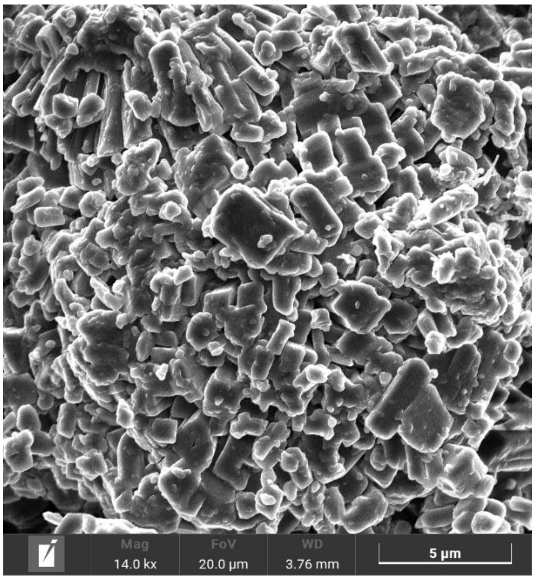

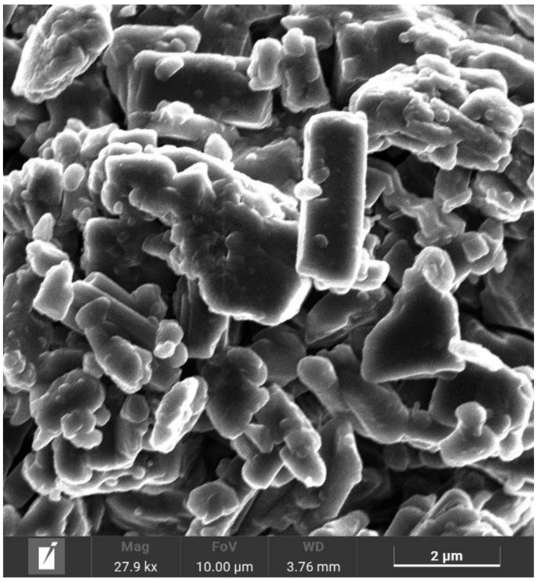

Figure S2. SEM images of GR-MOF-20 using axial detector.

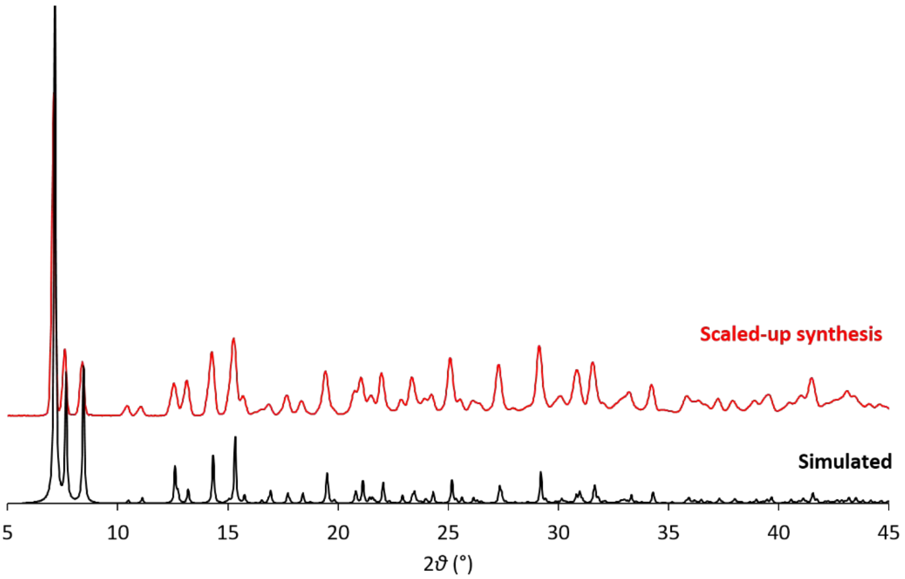

Figure S3. Powder X-ray diffraction (PXRD) patterns of the scaled synthesis of GR-MOF-20 (red) compared with single-crystal data (black).

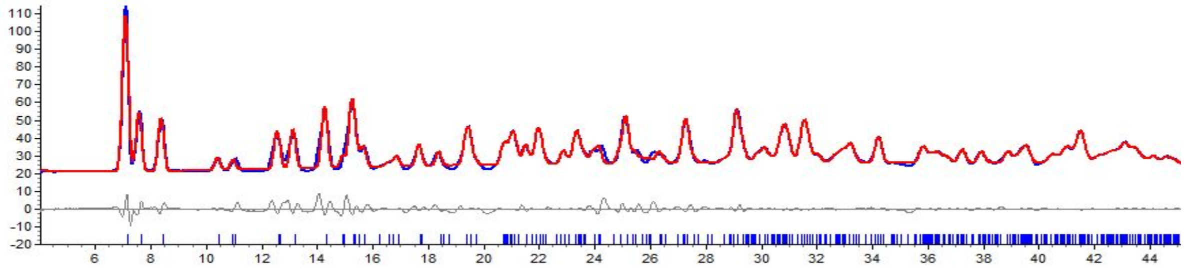

Figure S4. Le Bail fitting of GR-MOF-20.

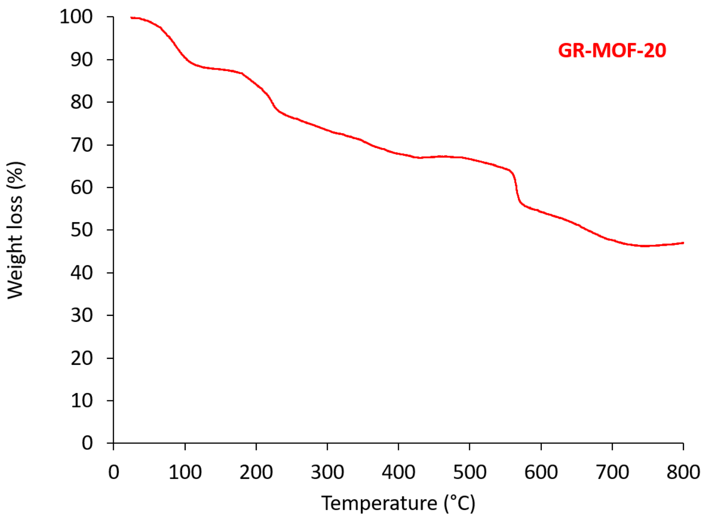

Figure S5. Thermogravimetric analysis (TGA) of GR-MOF-20.

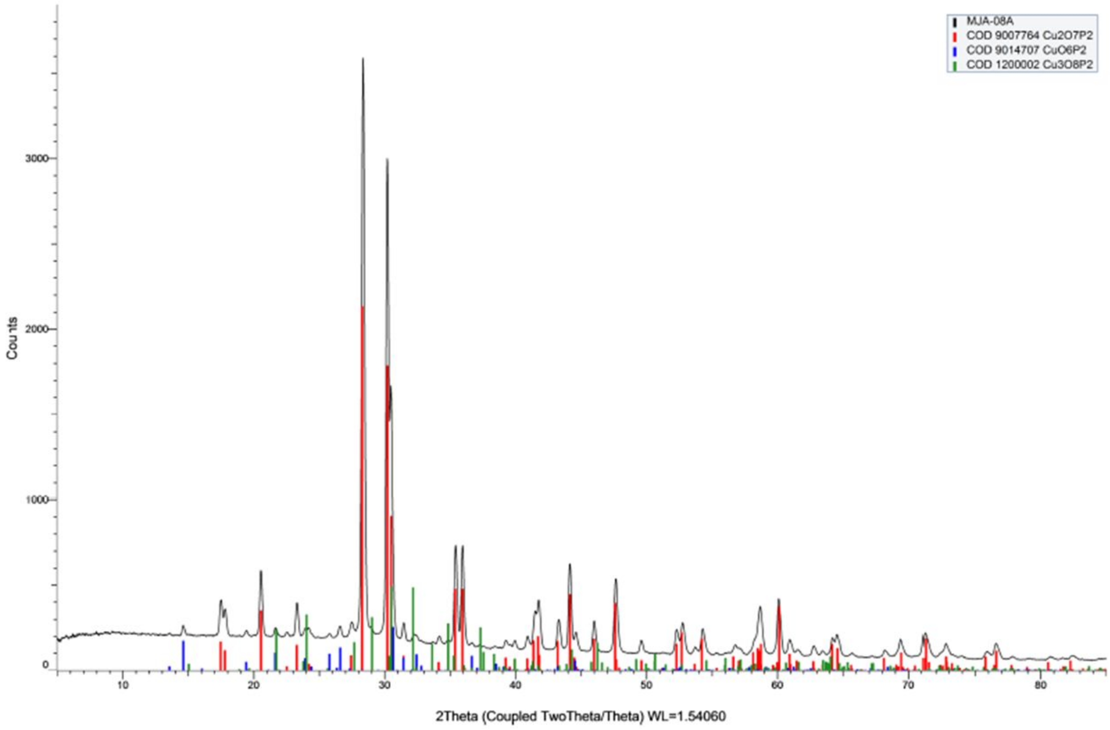

Figure S6. PXRD patterns of GR-MOF-20 after being heated up to 800 ˚C under air, and its identified residual species $Cu_3O_8P_2$, CuO_6P_2, and $Cu_2O_7P_2$.

S3. Stability studies

Quantification of 4,4'-Bipy by HPLC

4,4'-Bipy was determined using an HPLC system Shimadzu, equipped with a UV-vis detector SPD-M40 and autosampler SIL-40C controlled by LabSolutions software (Shimadzu). A Shim-pack GIS reverse-phase column (4 µm, 4.6 x 150 mm, Shimadzu) was employed. The mobile phase consisted of water (H_3PO_4, 0.1%) and acetonitrile 90:10. The analysis was carried out in isocratic mode and the injection volume was set at 10 µL with a flow rate of 0.8 mL·min^{-1}. The column temperature was fixed at 40 °C. The chromatograms of standard solution showed a retention time of 1.8 min corresponding to 4,4'-Bipy with absorption maximum (λ_{max}) at 250 nm.

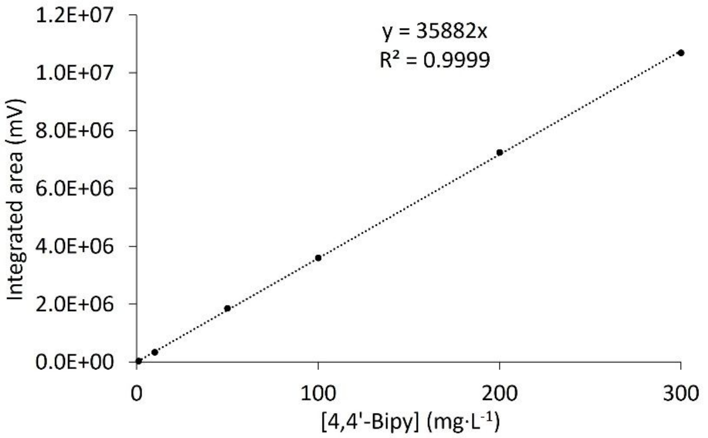

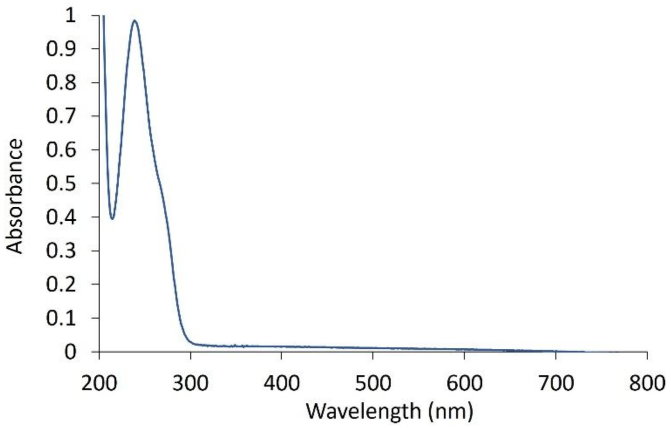

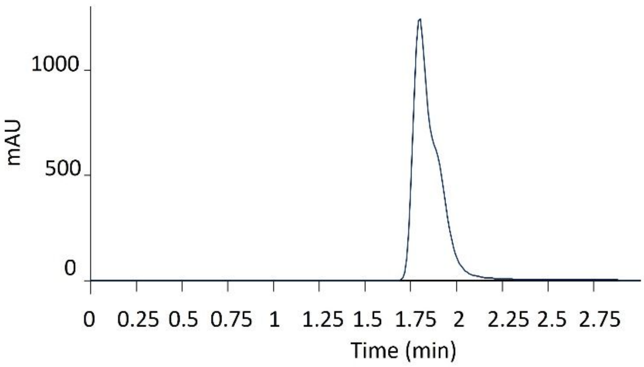

Figure S7. Calibration plots, UV-vis absorption spectra and chromatogram of 4,4'-Bipy in water by HPLC method.

S4. Herbicide activity

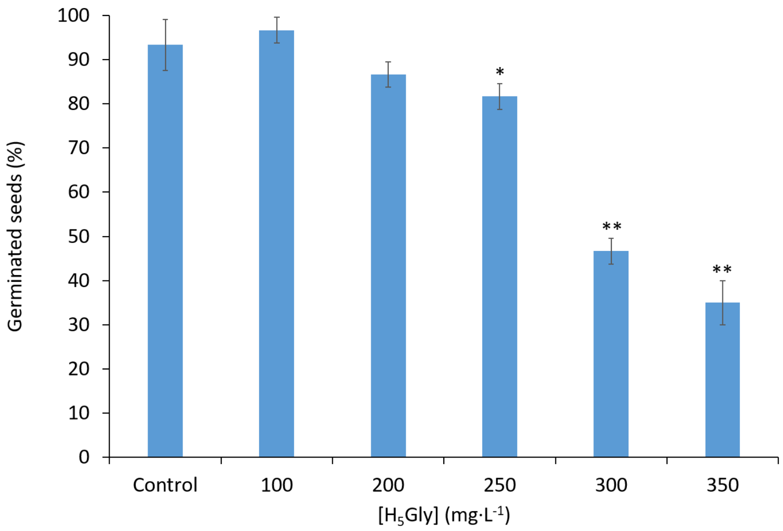

Figure S8. Germinated seeds (%) after being treated with aqueous solutions containing different concentrations of H_5Gly and water (control) for 7 days. Each experiment was assessed with *ca.* 20 seeds, where the average and the standard deviation are represented. The statistical significance with respect to the control was disclosed as $^{*}p < 0.05$; $^{**}p < 0.01$.

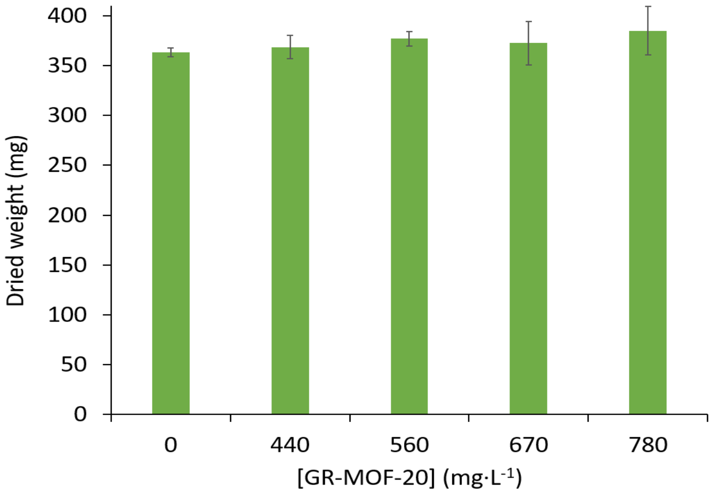

Figure S9. Effect of different concentration of GR-MOF-20 on growth (dried weight) on the non-target plant (wheat) seedling for 5 days.

S5. Evaluation of the antibacterial activity

Table S2. Determination of *E. coli* bacterial viability by plate count after 24 h in contact with GR-MOF-20 suspensions, and $Cu(NO_3)_2{\cdot}3H_2O$, H_5Gly, and 4,4'-Bipy solutions, and water as controls.

Sample	Concentration ($mg{\cdot}L^{-1}$)	$CFU{\cdot}mL^{-1}$	Inhibition (%)	Log_{10} ($CFU{\cdot}mL^{-1}$)
Control	0	$1.6{\cdot}10^9$	0.00	9.20 ± 0.07
GR-MOF-20	450	$1.8{\cdot}10^5$	99.98	4.97 ± 0.67
$Cu(NO_3)_2{\cdot}3H_2O$	275	$3.3{\cdot}10^4$	99.99	4.51 ± 0.1
H_5Gly	200	$5.7{\cdot}10^8$	64.37	8.75 ± 0.8
4,4'-Bipy	120	$5.7{\cdot}10^8$	64.37	8.75 ± 0.13

Table S3. Determination of *P. syringae* viability by plate count after 24 h in contact with GR-MOF-20 suspensions, and $Cu(NO_3)_2{\cdot}3H_2O$, H_5Gly, and 4,4'-Bipy solutions, and water as controls.

Sample	Concentration ($mg{\cdot}L^{-1}$)	$CFU{\cdot}mL^{-1}$	Inhibition (%)	Log_{10} ($CFU{\cdot}mL^{-1}$)
Control	0	$4.8{\cdot}10^8$	0	8.66 ± 0.19
GR-MOF-20	450	0	100	-
$Cu(NO_3)_2{\cdot}3H_2O$	275	0	100	-
H_5Gly	200	$6.2{\cdot}10^8$	0	8.79 ± 0.07
4,4'-Bipy	120	$1.6{\cdot}10^8$	66.67	8.19 ± 0.07

Table S4. Antibacterial activity of previously reported Cu-MOFs. All data are referred to previous experiments with *E. coli*.

MOF	Concentration or amount	Inhibition test	Incubation period (h)	Reference
GR-MOF-20	445 $mg{\cdot}L^{-1}$	CFU – 99.98% inhibition	24	Present work

GR-MOF-7	2.5 mg·L^{-1}	CFU – 100% inhibition	20	[4]
Cu-BTC	1 cm MOF disk	Inhibition halo – 4 mm	48	[5]
Cu-GA	1 cm MOF disk	Inhibition halo – 17 mm	48	[5]
Cu-MOF-1; 2; 3; 4	20 mg·L^{-1}	CFU – 99.9% inhibition	24	[6]
Cu-MOF	50 mg	Inhibition halo – 39.09 mm	24	[7]
Cu-MOF (H2BTC)	250 mg·L^{-1}	Inhibition halo – 20 mm	24	[8]
IEF-23	2 mg	Inhibition halo – 5.4 mm	24	[9]

S6. References

(1) Sheldrick, G. M. SHELXT – Integrated Space-Group and Crystal-Structure Determination. *Acta Crystallogr. Sect. A Found. Adv.* **2015**, *71* (1), 3–8. https://doi.org/10.1107/S2053273314026370.

(2) Sheldrick, G. M. Crystal Structure Refinement with SHELXL. *Acta Crystallogr. Sect. C Struct. Chem.* **2015**, *71* (1), 3–8. https://doi.org/10.1107/S2053229614024218.

(3) Dolomanov, O. V.; Bourhis, L. J.; Gildea, R. J.; Howard, J. A. K.; Puschmann, H. OLEX2 : A Complete Structure Solution, Refinement and Analysis Program. *J. Appl. Crystallogr.* **2009**, *42* (2), 339–341. https://doi.org/10.1107/S0021889808042726.

(4) Sierra-Serrano, B.; García-García, A.; Hidalgo, T.; Ruiz-Camino, D.; Rodríguez-Diéguez, A.; Amariei, G.; Rosal, R.; Horcajada, P.; Rojas, S. Copper Glufosinate-Based Metal–Organic Framework as a Novel Multifunctional Agrochemical. *ACS Appl. Mater. Interfaces* **2022**, *14* (30), 34955–34962. https://doi.org/10.1021/acsami.2c07113.

(5) Elmehrath, S.; Ahsan, K.; Munawar, N.; Alzamly, A.; Nguyen, H. L.; Greish, Y. Antibacterial Efficacy of Copper-Based Metal–Organic Frameworks against Escherichia Coli and Lactobacillus. *RSC Adv.* **2024**, *14* (22), 15821–15831. https://doi.org/10.1039/D4RA01241K.

(6) Jo, J. H.; Kim, H.-C.; Huh, S.; Kim, Y.; Lee, D. N. Antibacterial Activities of Cu-MOFs Containing Glutarates and Bipyridyl Ligands. *Dalt. Trans.* **2019**, *48* (23), 8084–8093. https://doi.org/10.1039/C9DT00791A.

(7) Xu, X.; Ding, M.; Liu, K.; Lv, F.; Miao, Y.; Liu, Y.; Gong, Y.; Huo, Y.; Li, H. The Synthesis and Highly Effective Antibacterial Properties of Cu-3, 5-Dimethy l-1, 2, 4-Triazole Metal Organic Frameworks. *Front. Chem.* **2023**, *11*, 1124303. https://doi.org/10.3389/fchem.2023.1124303.

(8) Ameen, R.; Rauf, A.; Mohyuddin, A.; Javed, M.; Iqbal, S.; Nadeem, S.; Aroosh, K.; ur Rehman Aziz, A.; Alhujaily, A.; Althobiti, R. A.; Alzahrani, E.; Farouk, A.-E.; Al-Fawzan, F. F.; Elkaeed, E. B. Excellent Antimicrobial Performances of Cu(II) Metal Organic Framework@Fe3O4 Fused Cubic Particles. *J. Saudi Chem. Soc.* **2023**, *27* (6), 101762. https://doi.org/10.1016/j.jscs.2023.101762.

(9) Lelouche, S. N. K.; Albentosa-González, L.; Clemente-Casares, P.; Biglione, C.; Rodríguez-Diéguez, A.; Tolosa Barrilero, J.; García-Martínez, J. C.; Horcajada, P. Antibacterial Cu or Zn-MOFs Based on the 1,3,5-Tris-(Styryl)Benzene Tricarboxylate. *Nanomaterials* **2023**, *13* (16), 2294. https://doi.org/10.3390/nano13162294.
